# Foci on breast magnetic resonance imaging in high-risk women: cancer or not?

**DOI:** 10.1007/s11547-016-0644-3

**Published:** 2016-05-12

**Authors:** Paola Clauser, Enrico Cassano, Arianna De Nicolò, Anna Rotili, Bernardo Bonanni, Massimo Bazzocchi, Chiara Zuiani

**Affiliations:** Institute of Diagnostic Radiology, Department of Medical and Biological Sciences, University of Udine, P.le Santa Maria della misericordia, Udine, 33100 Italia; Department of Biomedical Imaging and Image-guided Therapy, Division of Molecular and Gender Imaging, Medical University of Vienna, Waehringer Guertel 18-20, 1090 Vienna, Austria; Division of Breast Radiology, European Institute of Oncology, Via G. Ripamonti 435, 20141 Milan, Italy; Radiology Department, APSS-Trento, Via Degasperi 79, 38123 Trento, Italy; Division of Cancer Prevention and Genetics, European Institute of Oncology, Via G. Ripamonti 435, 20141 Milan, Italy

**Keywords:** Breast, Magnetic resonance imaging, BI-RADS, High-risk, Cancer

## Abstract

**Purpose:**

To assess how frequently foci are identified on MRI in high-risk patients, and their association with malignancy, breast density, and background parenchymal enhancement (BPE).

**Materials and methods:**

In this multicentric study, two readers, in consensus, retrospectively reviewed screening breast MRI of 245 high-risk women, performed between 2009 and 2014. Eligible patients had at least two consecutive screening MRI, and a follow-up of at least 1 year after a lesion was first detected; histology was available for all suspicious findings. Breast density, BPE (both using BI-RADS lexicon), presence, and changes at follow-up for foci were evaluated. Clinical history of the patients was reviewed. Chi-square test was used to define significant correlations.

**Results:**

166 women (mean age 43 years), who underwent a median of 4 MRI (range 2–6) during the study period, were included. 68 foci were found in 58 women [34.9 %, 95 % confidence interval (CI) 28.1–42.5 %]. Foci were more frequent in dense breasts (*P* = 0.079) and with moderate or marked BPE (*P* < 0.001). During follow-up, two foci increased in size (2.9 %, 95 % CI 0.8–10.1 %) and at biopsy, a cancer was found (1 high-grade ductal carcinoma in situ, 1 tubular carcinoma). Breast cancer was diagnosed in the other three cases, not initially appearing as foci, and it was more frequent in women with dense breasts (*P* = 0.04); no correlation between cancer and BPE was found (*P* = 0.145).

**Conclusions:**

Foci are relatively frequent in screening MRI, and they are usually benign. An increase in size is the most reliable criteria to suspect malignancy.

## Introduction

High-risk women present a lifetime risk of developing breast cancer higher than 20 % [1]. This increased risk is related to several factors, the more relevant being: the presence of a gene mutation (BRCA 1 and BRCA 2 being the more common), family history of breast or ovarian cancer (the first-degree relatives, with an early onset of the disease) [1, 2]. Malignant lesions found in these women are characterized by an early onset and by a high proliferation rate, thus being often more aggressive, as compared to the cancer usually diagnosed in the general population [3, 4].

In consideration of this evidence, various dedicated screening programs have been developed to allow early diagnosis in high-risk patients. These programs start at a young age, usually 30 years old or as soon as the risk factor is found. Along with the traditional imaging modalities, such as mammography and ultrasound, breast magnetic resonance imaging (MRI) plays a central role [1, 5]. Breast MRI has the highest sensitivity in breast cancer detection [6]; several multicentric studies proved that MRI, compared to mammography and ultrasound, is able to identify a higher number of cancers and at an earlier stage [7–10].

MRI is also able to detect very small enhancing lesions, with 5 mm or lower maximum diameter, which might be difficult to further characterize. These small lesions are defined by the American College of Radiology Breast Imaging Reporting and Data System (ACR BI-RADS) as foci. A focus is a small dot of enhancement that stands out from parenchymal enhancement. Per definition, foci cannot be accurately assessed with respect to margin or internal enhancement: if these characteristics can be assessed, the finding should be considered a small mass [11]. Foci are frequently associated with an increased hormonal stimulation, and they can sometimes be seen when a benign lesion is present (fibroadenoma, cyst and fibrocystic changes, lymph node), but they can also represent the early onset of a malignant lesion [12, 13]. Studies addressing the malignancy rate of foci found in the general population showed highly variable results, with percentages ranging from 0.6 to 23 % [12, 14]. Thus, the best management of foci is still under discussion. The issue is of particular interest in high-risk women, especially considering the importance of early diagnosis in this group of patients. Despite this, not many studies addressed the frequency of foci detected during screening MRI in high-risk patients and the malignancy rate of foci in this population.

The aim of our study was to determine how frequently foci are identified on breast MRI in high-risk patients, and how frequently foci are found to be malignant. We further correlated the presence of foci and malignancy with breast density and background parenchymal enhancement.

## Materials and methods

### Patients’ collection

This retrospective study involved two institutes, both with dedicated breast units. Patients included gave their informed consent in both centres, and IRB approval was granted. In both institutions, women with family history of breast and ovarian cancer (more than one close relative, at a young age) are sent to genetic counselling. The risk and the likelihood of detecting a pathogenic gene change are calculated using a standard risk assessment modality (such as CaGene), and when deemed necessary, the patient undergoes genetic testing.

Screening is performed with annual breast MRI and ultrasound; digital mammography is performed annually in women older than 35 years. All breast MRIs performed for screening in high-risk women between January 2009 and October 2014 were reviewed.

Patients were included in this retrospective analysis when: at least two rounds of MRI screening were available; at least 1 year follow-up was available after a focus was detected for the first time; histological verification or follow-up of at least 1 year for all findings classified as BI-RADS 3 or higher was available. Cases that did not meet the inclusion criteria, incomplete MRI examinations, cases with previous bilateral mastectomy, and cases for which information about risk factors was not available, were excluded.

The high-risk databases of the two institutions were reviewed to collect data on gene mutation and family history for the included patients. A total of 245 high-risk women were retrieved, and 169 met the inclusion criteria. Age at the time of first examination ranged between 23 and 68 years old (mean 43.6 years old).

### Breast MRI acquisition

In both the institutes, breast MRI was performed according to the guidelines defined by the EUSOMA working group [15], on 1.5-T magnet (Magentom Avanto, Siemens, Erlangen, Germany in both institutes), using vendor-supplied dedicated bilateral breast coils (four channels). Examinations were performed with patient in the prone position, among the 7th and 14th day of the menstrual cycle for pre-menopausal women. The standard protocol used for the clinical evaluation consisted of an axial Short-Tau Inversion Recovery (STIR) T2-weighted sequence and an axial spoiled Gradient-Echo 3D (FLASH) T1-weighted sequence acquired before and five times after the injection of contrast material for the dynamic study (Gadobenate Dimeglumine, Multihance, Bracco; 0.01 mmol/kg of body weight, injected at the rate of 2 ml/s, followed by a flush of 20 ml of saline solution). Technical parameters of the T1-weighted fast low-angle shot sequences were: TR 9 ms, TE 4.76 ms, FOV 340 × 340 mm, slice thickness 2 mm, matrix 512 × 512 at one institution; and 7.4, 4.7 ms, 340 × 340 mm, 1.3 mm, 384 × 369 at the other institution. Starting from 2012, an axial echo-planar imaging (EPI) diffusion-weighted sequence was also acquired.

### Image analysis

Two readers with more than 3 years of experience in breast imaging and breast MRI reviewed the images in consensus. Readers were aware of the indication for the MRI (screening), but they were blinded to the number and type of lesions present in the data set.

For each MRI examination, readers had to evaluate presence or absence or foci. The BI-RADS definition of a focus was strictly followed [11], and lesions that could be further characterized by evaluating pre- and post-contrast T1w sequences, T2w sequences, or DWI were not considered, even when presenting a diameter equal or lower to 5 mm (i.e., cysts, small spiculated masses, and lymph nodes). In patients showing strong, punctate, background enhancement, a focus was described only when showing enhancing characteristics clearly different from that of the remaining fibroglandular tissue. For all detected foci, readers had to state whether in the subsequent examinations, the lesion was disappearing, reducing in size, stable, increasing in size, or showed any change in morphology suggesting malignancy. Number of foci per patient was also considered.

Breast density was evaluated on pre-contrast T1w sequences and classified according to the BI-RADS lexicon: (a) almost entirely fat; (b) scattered fibroglandular tissue; (c) heterogeneous fibroglandular tissue; and (d) extreme fibroglandular tissue. Pattern of background parenchymal enhancement (BPE) was defined on the post-contrast study, according to the BI-RADS lexicon: (a) minimal; (b) mild; (c) moderate; and (d) marked.

### Statistical analysis

Overall number of newly appearing foci was calculated. Malignancy rate for foci was calculated as number of breast cancers initially appearing as foci as compared to overall number of foci. Proportions are presented as percentages with 95 % confidence interval (95 % CI).

The relation between presence of foci in patients with different breast densities or in MRI with different BPEs was evaluated using the Chi-square test.

Finally, the presence of malignant lesions was compared in patients with or without foci, and considering breast density and BPE, using the Chi-square test. Analysis was performed using the statistical software commercially available (MedCalc Software v.20, Ostend, Belgium).

## Results

Three patients were excluded from the evaluation, because a malignant lesion was diagnosed during the first MRI examination.

Overall, 166 patients were included, and 640 MRI examinations were performed, with a median of four examination per patient (range 2–6 examinations).

Of these 166 patients: 100 had a known pathologic mutation (46 BRCA 1, 52 BRCA 2, 1 BRCA 1 and 2, 1 p53); 17 were tested and the results were negative for BRCA1 and BRCA2 mutations (BRCAX); and 49 had a calculated risk superior to 20 %, but were not tested or results were not available at the time of data collection.

At least one focus was detected in 52/166 (31.3 %, 95 % CI 24.8–38.7 %) patients during the first MRI examination and in other six patients (3.6 %) in a subsequent examination, overall foci were found in 58/166 patients (34.9 %, 95 % CI 28.1–42.5 %). A single focus was found in 50 patients (86.2 %, 95 % CI 75.1–92.8 %), while two or more foci were detected in the remaining eight patients (13.8 %, 95 % CI 7.2–24.9 %). Overall, 68 foci were detected. When considering separately each MRI, one or more focus was detected in 215/640 examinations (33.6 %, 95 % CI 30.0–37.3 %).

During follow-up, the majority of the foci were stable: 57 foci showed no changes in dimensions or morphology (83.8 %, 95 % CI 73.3–90.7 %). Nine foci disappeared during follow-up (13.2 %, 95 % CI 7.1–23.3 %), 1 or 2 years after the examinations where they were first detected. In none of the cases, a reduction in size was clearly visible.

**Fig. 1:**
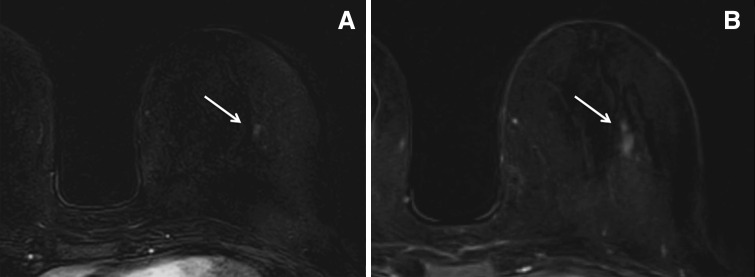
A small focus was detected in the central area of the left breast in a screening MRI performed in a 46-year-old woman (**a**, *arrow*). The finding was considered non suspicious and the patient was sent to 1-year control. After 1 year (**b**, *arrow*), the area increased in size and MR-guided biopsy was performed. Histology showed a high-grade ductal carcinoma in situ

**Fig. 2:**
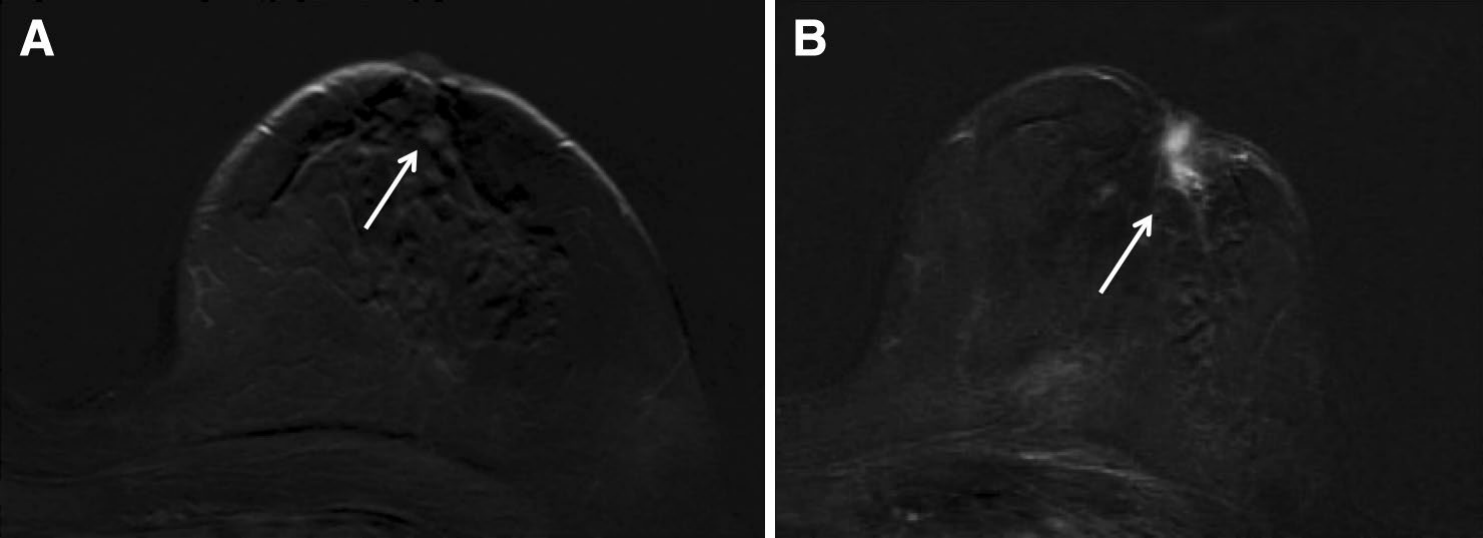
A small focus was detected in the retroareolar region of the left breast in a screening MRI performed in a 62-year-old woman (**a**, *arrow*). The finding was considered non suspicious and the patient was sent to 1-year control. After 1 year (**b**, *arrow*), the patient came at the control with nipple retraction. A retroareolar mass enhancement was found, and a biopsy was performed under the U.S. guidance after the second-look ultrasound. Histology showed a tubular carcinoma

One focus (1.5 %) in a BRCA 2 mutate woman, after 1 year, increased from 5 to 10 mm, and showed also alterations in the morphology, appearing as a non-mass lesion (Fig. 1). One focus (1.5 %) in a non-tested woman with a strong family history increased from 5 to 15 mm at 1 year follow-up. Also in this case, a slight modification in morphology was found, and a mass lesion on T1-weighted sequences could be seen (Fig. 2). In both the cases, the lesion was biopsied and histology was high-grade ductal carcinoma in situ and tubular carcinoma, respectively. Malignancy rate for foci was 2.9 % (2 on 68, 95 % CI 0.8–10.1 %).

Other three cancers were found during the study period in one BRCA 2 patient and in two non-tested patients: two invasive ductal carcinomas and one high-grade ductal carcinoma in situ. Two high-risk lesions were also detected in other two patients: one phyllodes tumour and one atypical ductal hyperplasia. Neither the other cancers nor the high-risk lesions initially appeared as a focus. Overall, 2 on 5 cancers detected during the study period (40 %) were initially visible as foci, without any further suspicious characteristic.

**Fig. 3:**
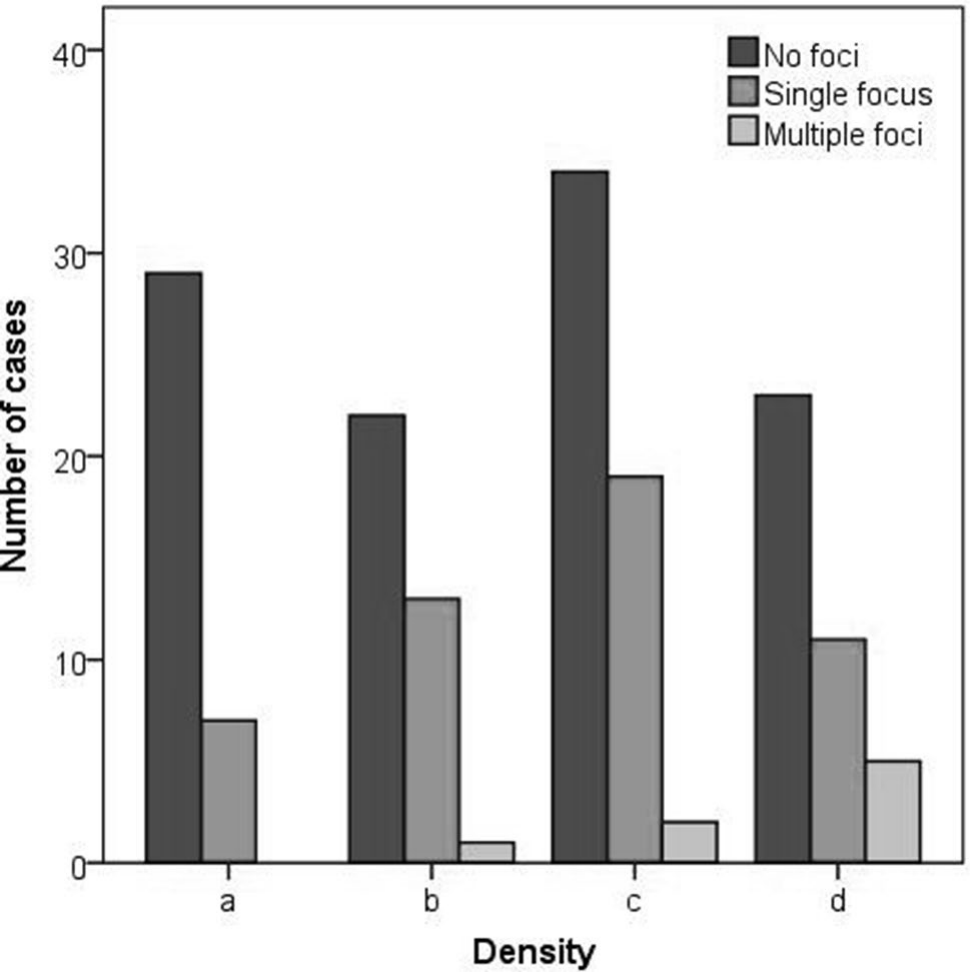
Distribution of foci according to breast density, classified using the BI-RADS lexicon

Breast density distribution and distribution of foci and malignancy in the various density classes are shown in Table 1. No significant difference in the distribution of foci was found (*P* = 0.079, Fig. 3), though a trend towards a higher percentage of foci in dense breast was detected. A significantly higher number of cancers were detected in patients with dense breasts compared to those with non-dense breasts (*P* = 0.04). A similar result was obtained when considering both cancers and high-risk lesions (*P* = 0.03).

**Table 1:** Number of patients presenting with foci, number of foci, and cancer cases according to breast density distribution

Breast density	Patients with foci		Foci	Cancer cases
	Single focus	Multiple foci	*N*	*N*
*a* = 36	7 (19.4)	0 (0.0)	7	0
*b* = 36	13 (36.1)	1 (2.8)	15	1
*c* = 55	19 (34.5)	2 (3.6)	24	4
*d* = 39	11 (28.2)	5 (12.8)	22	0

Percentages are given in brackets

Distribution of BPE, foci, and malignant lesions is shown in Table 2.

**Table 2:** Number of foci and cancer cases according to background parenchymal enhancement distribution

Background enhancement	Number of foci *N* (%)	Cancer cases *N*
*a* = 458	151 (32.9)	2
*b* = 117	57 (48.7)	3
*c* = 44	35 (79.5)	0
*d* = 21	7 (33.3)	0

**Fig. 4:**
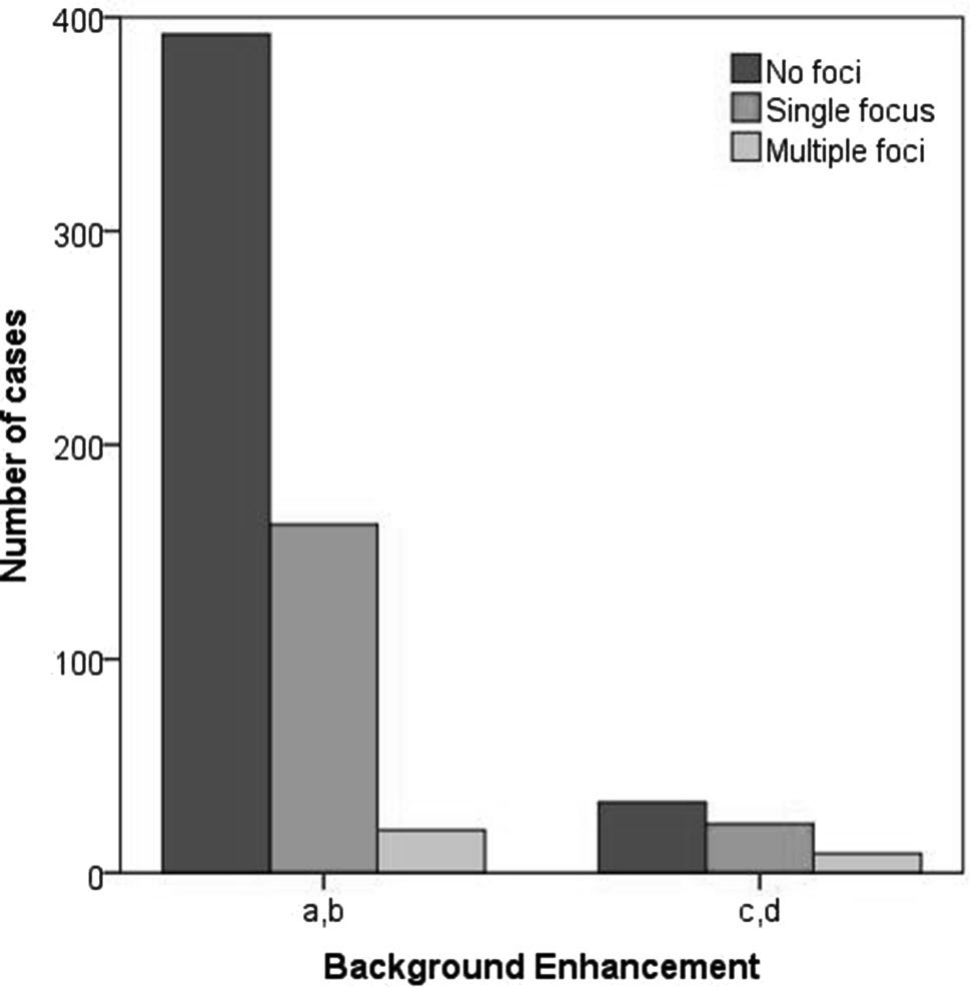
Distribution of foci according to breast background parenchymal enhancement (BPE), in patients with minimal or mild BPE (ACR BI-RADS *a*, *b*) and moderate or marked BPE (ACR BI-RADS *c*, *d*)

Foci were more frequently encountered when BPE was present (*P* < 0.001), and this significant difference was confirmed also when comparing low (a and b) with high (c and d) levels of BPE (*P* < 0.001, Fig. 4). There was no difference in cancer distribution related to BPE (*P* = 0.145), and the same was found when considering also high-risk lesions (*P* = 0.328).

## Discussion

We identified foci in one-third of breast MRI screening examinations performed during a 5-year period in high-risk patients. Only in the two cases, these small areas of non-specific enhancement were found, at follow-up, to be malignant lesions (2.9 %). Malignancy was more frequent in dense breasts, but no correlation with BPE was found.

Foci on breast MRI can be associated with hormonal stimulation or with a wide variety of findings, being most often related to benign proliferative or non-proliferative changes of the breast, cysts, or small lymph nodes. Rarely, they might represent an early sign of malignancy.

Foci were detected in a higher number of patients, as compared to other studies [14, 16]. This difference might have various explanations: high number of young patients with dense breast, thus with a higher hormonal stimulation, the different definitions of focus used in different studies, and the different acquisition parameters and thus different capabilities of distinguish a true focus from a small lesion. Of note, we obtained a malignancy rate well within the lower limits of the range presented in the literature for the general population [12, 14]. There is a significant variability regarding the malignancy rate of foci (from 0.6 to 23 %) [12–14, 17–21]. This high variability can have various explanations the more relevant being case selection and the definition of focus. In some studies [14, 21, 22], lesions visible on pre-contrast T1w or T2w sequences, and thus amenable of further characterization, were classified as foci on the basis of their maximum diameter. According to the latter BI-RADS definition [11], this category of MRI findings was excluded from our analysis. The highest spatial resolution achievable with new sequences and higher field strengths allows obtaining more details on lesions characteristics, and it is mandatory to carefully evaluate the small areas of enhancement in all sequences, as important diagnostic information can be detected [23].

Liberman et al. [12] analysed a small group of high-risk patients and found a similar percentage of malignancy in their evaluation, also not different from the malignancy rate of the general population.

Management of foci is still a topic of discussion. According to most of the literature, foci might represent cancer in more than 2–3 % of the cases, and thus in specific single cases, (synchronous breast malignancy, patients’ decision) biopsy should be considered [24]. On the other hand, it is highly unlike that the decision to biopsy all foci would be cost-effective, as in most of the cases, biopsy can only be performed under MR-guidance and the majority of the lesions will turn out to be benign [17, 24]. The only sign on which various studies agree to define a focus as suspicious is the increase in size [24]; thus, indication for biopsy should be given only when an increased focus is found. Furthermore, modification in the appearance of the focus, related or not to a modification in size, should be considered suspicious and indicate the need for a biopsy. In both our cases, cancers detected showed a minimal growth at follow-up after 1 year. As 6 months control was not performed, it is not possible to state whether it would have allowed an earlier diagnosis. Short-term follow-up could help in the earlier detection of lesions increasing in size but, on the other hand, a small increase might be undetected when performing a short-term evaluation. Of note, 2 on 5 cancer diagnosed were initially visible as a focus, thus suggesting the need to identify more features helpful in the early characterization of foci.

Though not many data are available on the effect of BPE on lesion identification on MRI, it is likely that benign proliferative changes in the breast can be associated to both foci and BPE [25].

Several studies already showed the importance of breast density as a risk factor for breast cancer [26], and these were also seen in our study. A recent study [27] found a correlation between BPE and cancer risk, but this was not confirmed by our results. The relatively small number of cases included in our analysis might have limited this evaluation.

Our study has some limitations: though two centres were involved, the overall number of foci and cancer detected was not high and it is not possible to draw conclusions on management. In addition, though average follow-up was 4 years, many patients underwent only a 2-year follow-up. Especially in the beginning of the study period, several patients were lost at follow-up or did not agree to participate in the study. We believe this was strongly related to poor patients’ awareness and scarce implementation of structured national screening programs. Increased information to patients and more structured programs can improve women’s compliance to MRI screening. An overall small number of cancers were found during the analysed period. We believe that this result is mainly related to our case selection: some cancer cases were not included in the study, as not enough follow-up was available. Furthermore, three patients initially included in the analysis were then excluded as cancer was diagnosed at the first examination. These patients were excluded as the modifications related to the therapy might have affected image interpretation.

In conclusion, we found that foci are a relatively frequent finding in screening breast MRI performed in high-risk women, but they are rarely related to malignancy. Malignant lesions were more frequent in women with dense breast, while no relation with background parenchymal enhancement was found. Malignancy rate of foci does not seem to be higher, as compared to the general population, thus suggesting that the same management could be adopted. When a focus increases in size, or shows suspicious imaging characteristics, biopsy must always be performed. Whether the best management of a newly detected focus is biopsy, short-term follow-up or 1-year follow-up is still under discussion; and further studies will be necessary to clarify this issue.
